# ‘Hold the course(s)!’ – a qualitative interview study of the impact of pandemic-triggered contact restrictions on online instruction in community-based family medicine teaching

**DOI:** 10.3389/fmed.2023.1231383

**Published:** 2023-08-02

**Authors:** Marie-Theres Steffen, Hannah Fuhr, Stefanie Joos, Roland Koch

**Affiliations:** Institute for General Medicine and Interprofessional Health Care, Tuebingen, Germany

**Keywords:** digital transformation, family medicine teaching, grounded theory, medical education, online education, online instruction, pandemic

## Abstract

The COVID-19 pandemic has been identified as a catalyst for the digitalization of medical education. Less is known about the specific impact of the pandemic on decentralized, community-based education, such as in General Practitioner practices. The aim of this study is to understand the impact of the digital transformation process, triggered by the COVID-19 pandemic. As, family medicine courses involve both university-based and community-based teaching, this study focuses the mode and quality of instruction and instructors in family medicine teaching. A qualitative interview study was conducted. The participants (*N* = 12) of a multi-perspective Quality Circle in family medicine teaching were interviewed twice: first, in 2019, about digitalization in family medicine teaching in Tübingen, Germany, not yet aware of the global changes and local transformation that would take place shortly thereafter. Second, in a follow-up interview in 2020 about the transition process and digitalization following the impact of contact restrictions during the pandemic. Grounded Theory was used as a qualitative research approach to analyze the complex processes surrounding this transformation. By analyzing the interviews with various stakeholders of community and university-based teaching, a model for the digital transformation process of family medicine teaching at the University of Tübingen in response to an external stimulus (the pandemic) was developed. It involves six chronological steps: “The calm before the storm,” “The storm hits,” “All hands on deck,” “Adrift,” “Reset course,” and “The silver lining.” This model seeks to understand the process of digital transformation and its impact on the teaching institution (medical faculty of the University of Tübingen, Institute for General Practice and Interprofessional Health Care) and instructors from an integrated perspective and thereby critically revisits prior concepts and opinions on the digitalization of medical teaching. Insights gained are presented as key messages.

## Introduction

With COVID-19’s effects on nearly all aspects of society, the pandemic’s impact on medical teaching was not in the headlines. However, profound effects on medical teaching were observed worldwide as contact restrictions in many countries led to a transition from in-seat teaching to mostly online instruction (1–3).

Already prior to the pandemic, the use of online instruction in medical education had become more common (4, 5). In most universities in Germany, some online instruction had been implemented before the COVID-19 pandemic but was mostly restricted to individual pilot projects and thus heterogeneous (6). Among other reasons, lecturers’ lack of experience with online instruction methods and uncertainties regarding data protection regulations played a role in the nationwide low level of implementation (7, 8).

With the transformation processes during the COVID-19, roles and responsibilities of medical educators (General Practitioners (GP) teachers, other teachers employed at the university for teaching, teaching coordinators, and supporting staff) changed to include new aspects, such as moderating video conferences, and creating or distributing meaningful digital content, such as podcasts (9). Various authors reported their initial concerns were reduced after using digital formats (10–12). Other concerns, such as the difficulty of achieving meaningful feedback without face-to-face contact, were confirmed (13–15).

These concerns address key elements of instructional communication and teaching competencies, which include both the subject knowledge and the ability to communicate that knowledge engagingly. Being able to elicit attentiveness, emotional engagement, and being able to process feedback given by students in the ensuing communication loop are further competencies of successful teachers (16, 17).

The overall experience of online instruction was described as enriching, and many aspects were found to be worthwhile maintaining to complement in-seat teaching (18–24). Concerning lecturers’ attitudes at a later stage in the pandemic, Dorfsman et al. identified three types: The enthusiasts who are interested in long-term change but do not get into specifics, the experienced ones who have substantially changed their teaching styles and plan to maintain online instruction in the future, and the critics who have adapted to the circumstances but yearn to return to the “normal” pre-COVID-19 teaching situation (25).

Students found that online instruction had the potential to support individual learning and promoted learner engagement (26–28). They evaluated the digital formats positively for the transfer of theoretical knowledge (29, 30) while also pointing out deficiencies in practical content and applicability to clinical practice (14, 15). The effects of online instruction on the learning process were rated overall as beneficial (31, 32). During the COVID-19 pandemic, the understanding of medical students’ roles changed from the predominant role of learners to that of medical providers (33). As a result, students in their final years of schooling were integrated more quickly and intensively into the clinical routine. At the same time, less advanced students were denied access to practical training (2, 34). The burden on many medical students increased, especially in cases of pre-existing mental illnesses (35, 36) or with financial hardships (37–40).

### Digitalization in community-based teaching in family medicine – a blank spot on the map

Most research related to the digital transformation of teaching has focused on university-based teaching. Less attention has been paid to the digitalization of teaching in decentralized or community-based settings (e.g., clerkships in outpatient GP practices) (41) during the pandemic. Teaching in these settings presents complex challenges due to the independence of such environments from university-based teaching, the incorporation of various stakeholders, and the complex social interaction with those stakeholders (42–44). Before the pandemic, digitalization of teaching and quality management of decentral teaching formats in Germany had been identified as two major areas in need of improvement (45, 46). During the pandemic, only a few examples of online instruction in family medicine were published in Germany, such as the blended learning approach described by the family medicine department in Homburg (24, 47, 48).

In summary, little is known about how the transformation of digitalization during the pandemic affected community-based teaching, instructional communication and communication between university-based medical educators and community-based GP teachers. An integrated analysis of the perspectives of said stakeholders on the digitalization of medical teaching, especially under externally imposed restrictions, has hitherto not been considered in this area of interest.

### Aim of the study

The aim of the study is to derive a model for the digital transformation of family medicine teaching based on the experiences of stakeholders before and after the pandemic. Based on our model and lessons learned during the pandemic, the study aims to describe how to approach the digital transformation of community-based teaching formats in family medicine teaching.

## Methods

This qualitative interview study took place in two phases during 2019 (before the pandemic and contact restrictions) and 2020 (during the first semester under COVID-19 restrictions) at the Institute for General Practice and Interprofessional Health Care at the University of Tübingen in southern Germany. It follows the Standards for Reporting Qualitative Research (49).

### Setting

The Institute for General Practice and Interprofessional Health Care in Tübingen is part of a university hospital system in southern Germany. It cooperates with about 250 family medicine teaching practices located within a radius of 70 km around the city of Tübingen. During the first two weeks of each semester, 160 students complete a clinical clerkship in one of those practices (50). Before the COVID-19 pandemic, family medicine teaching in Tübingen was predominantly in-seat. The first online instruction formats had been planned prior to the pandemic and were to be piloted in the summer semester of 2020. The COVID-19 pandemic led to the following drastic restrictions on teaching: There was a general obligation to wear a mask. Bedside teaching was dropped. Group sizes were severely restricted due to distancing regulations. Consequently, many courses had to be digitized much sooner than originally planned, starting with the summer semester of 2020. The family medicine clinical clerkship in the community GP teaching practices was canceled. Other formats, like a physical examination course at the university, could be offered by using blended learning, simulation patients, and robust hygiene measures.

### Study design and data collection

The first round of interviews took place between October 16th, 2019 and November 15th, 2019 in the context of a study that examined the organization and function of a Quality Circle (QC) for family medicine teaching in Tübingen. A QC is a format in which participants meet regularly to discuss challenges and potential solutions related to a particular professional topic. The QC for family medicine teaching in Tübingen consists of relevant stakeholders in family medicine teaching, both from the university and community-based practice settings. In the QC study, individuals were interviewed about the structure and function of the QC in the context of an observed session on the digitalization of medical teaching at the university and in communities. The number of interview partners for the interview was limited by the number of participants in the QC (*N* = 13). All members of the QC except for MTS, who led the interviews (*N* = 12, 100%) agreed to participate in the first interview in 2019.

Starting in the summer semester of 2020, in-seat teaching had to be replaced almost entirely by online teaching due to the COVID-19 pandemic. To conduct the follow-up interviews on the transformation process in this changed situation, the interviewees from the first phase were contacted again. All but one of the prior interview partners (*N* = 11, 92%) took part in the follow-up interviews at the end of the first digital semester under COVID-19 restrictions from August 3rd, 2020 to October 3rd, 2020.

After providing informed consent to participate in the study, QC members were interviewed individually either in person or by telephone in the first interview phase. In the second interview phase all interviews were done by telephone due to contact restrictions. MTS conducted all interviews. A semi-structured interview outline was used for both rounds of interviews. The first interview of each phase was considered a pilot interview. It was reviewed by the author team regarding interview style, structure, and contents, leading to minor changes to the interview outline. Among other topics, such as the work processes and methods of the QC, the first interview outline explored the digitalization of family medicine. A translated version from the original German is included as a supplement (see Supplementary Table S1). In the second interview phase, the outline was expanded with questions about processes surrounding the online instruction that was taking place (for the translated outline, see Supplementary Table S2). The interviews were recorded using a digital audio recorder (Tascam DR-22WN), transcribed verbatim, and depersonalized using pseudonyms. During both interview phases the transcripts revealed a thematic saturation after nine (first phase) and ten interviews (second phase) concerning the digitization and transformation process. At that point, new codes no longer had to be added to the coding system but rather the data could be integrated into the existing coding scheme.

### Data analysis

The transcripts were analyzed with a Grounded Theory (GT) approach using the MAXQDA Software (VERBI Software GmbH, Berlin). Analysis was performed in three consecutive steps, as proposed by Strauss and Corbin (51). We chose GT as a methodological approach due to the lack of pre-existing literature and our aim of exploring the transition processes with open minds rather than preformed judgments.

The analysis process took place in three consecutive steps, beginning with open coding, in which the data material was broken into separated parts, carefully coded, and sorted into categories (51). The coding frame was developed on the basis of the first two interviews of the second interview phase. The coding frame was then discussed, adapted, and refined by RK and MTS. This coding framework was then used by MTS to code all remaining 21 interviews from both interview phases. After a break of at least four weeks, the interviews were coded again by MTS to ensure a high-density model. Discrepancies in coding processes were discussed within the author team and resolved through consensus building.

In the subsequent axial coding, cross-connections were formed between the categories using the proposed coding paradigm by Strauss and Corbin (51). Some important aspects in the transformation process were only mentioned retrospectively in the second interview phase. GT allows this to be included in the analysis, providing a missing consideration for the broader implementation of digital formats. In the last step, selective coding, the resulting axial codes were connected to each other in a more abstract way to encompass the entire data material in a core variable (51).

Within the research process, intermediate results were presented and discussed multiple times in an interdisciplinary research group workshop for qualitative methods led by a sociologist and qualitative researcher. The results were presented at a conference attended by GP teachers and university representatives and discussed there in the context of a peer-check (52). During these discussions among the authors and with colleagues experiencing digital transformation at other universities, a depiction with images and symbols from the nautical world arose and was deemed descriptive and illustrative. The following analysis refers to such images and metaphors where appropriate.

### Research team and reflection

Both the relationship between the interviewee and the researcher and the researcher’s engagement with the material may affect the analysis. Therefore, each author’s background will shortly be outlined (53): MTS is an assistant physician, a participant of the QC since 2019, and she wrote her dissertation on the QC. During the time of the interviews, MTS was a medical student. HF is a GP teacher with working experience in both German and international outpatient settings. SJ is a GP and head of the Institute of General Practice and Interprofessional Health Care at the University of Tübingen. RK is a GP and teaching coordinator, founder of the QC, and its moderator. As teaching coordinator, he was responsible for the digital transformation process at the Institute in Tübingen during the pandemic.

## Results

### Interview partners and population

The interview duration was 22 min, on average. The following Table 1 shows the characteristics of the interview partners sorted by gender, age, and profession. To ensure the anonymity of this small sample, the data is sorted separately.

**Table 1:** Characteristics of the interview partners.

Category	Item	Count (%)
Sex	Female	9 (75%)
	Male	3 (25%)
Age (year)	20–29	3 (25%)
	30–39	3 (25%)
	40–49	2 (17%)
	50–59	4 (33%)
Profession	Medical students	3 (25%)
	GP teachers	3 (25%)
	Course management and administration	3 (25%)
	Other	3 (25%)

### Open coding

Table 2 presents the main categories resulting from open coding: Perception of teaching, Transformation process, Future structuring, Comments about Quality Circle, and Communication.

**Table 2:** Codesystem.

Main category	Categories and subcategories level 1
Perception of teaching	Online instruction can do many things but not everything
	Impact of the transition on personal development
	What educators learn Different starting conditions of the interview partners
	Obstructive processes/problems Attentiveness reduced, difficult in online formats Cancellation of courses Practical content is insufficient Improvements are needed Lack of personal contact Burden of transition
	Advantages of online instruction Technology as an expansion of the teaching method Shows potential even for the discussion of difficult topics such as professionalism and emotional aspects of learning Digitization makes logistics easier Independence as regards location of learner and educator is considered positively
Process of transition	Special challenges in medical teaching
	Lack of uniform implementation by the faculty
	Exacerbation of social inequities
	Uncertainty at the beginning
	Dealing with technical aspects Guidance on technology provided by the institute There was no alternative to dealing with it
	Polarized opinions about digitization
	Pandemic as a driver of digitization
	Digitization is inevitable
	Adaptation of formats of projects Much tolerance/enthusiasm at the beginning Students appreciate free collaboration Surprisingly quick transformation Learning by doing in digitization Fast reaction required Teaching was adaptable Firmer rules of conversation in webinars needed
Future structuring	Digitization needs a clear goal
	Dependence on external circumstances
	Work load remains high
	Uncertainty is stressful
	Changes in university teaching Digitization is complex
	Digital formats should be adopted
	Educators are optimistic
	Feedback on digital formats needed
Comments about QC	QC has not played a major role
	Interest in other topics for QC
	Work of QC of the last months was/has been valuable
Communication	Sense of togetherness has been strengthened
	Communication structures need time
	Exchange among departments varies
	Poor accessibility due to home office
	Leadership was necessary
	Information came too late
	Exchange of information was cumbersome and slow

### Axial coding

Three codes were elaborated in the axial coding. The analysis results are documented with text passages, with ‘P_XX_19’ showing a quote from the first interviews in 2019 and ‘P_XX_20” a quote from the second interviews in 2020.

The first code, *The impact on those involved in the transformation towards online instruction*, describes the effects of the transition on individual stakeholders. For example, this included how interviewees with varying levels of experience coped with uncertainties of the pandemic environment and resulting restrictions. The experiences of the first digital semester also made interviewees abandon their initial preferred approach of slow, deliberate digital transformation as they realized it was not feasible. The transformation led to logistical advantages but also to increased social inequities and the loss of some central components of the curriculum, such as supervised professional development.

*P_08_19: “But I think the topic [of digitization] is still relatively far away from actual implementation, which makes it difficult to assess at this point.”*

*P_01_20: “And then it rather resulted that we had to train all our lecturers, […] in the shortest possible time.”*

The second code, *The transformation of university teaching*, addresses the transformation process on an institutional level (both at the university and in community-based teaching). Widespread implementation of online instruction would have been difficult to imagine in the first interview phase. During the transformation process, networking and organizational experience expanded. Many teaching formats turned out to be in need of improvement but surprisingly capable of change. The transition, hastened by the external force of COVID-19, was retrospectively viewed as major step.

*P_08_20: “So a lot happened, […] the exchange nationally and internationally has increased immensely for us during this time. […] I think that the knowledge has increased considerably.”*

The third code, *What educators learned,* contains separate codes that encompass the special role of educators in the transformation process. The rapid conversion to online instruction placed additional demands on educators, compounded by the fact that there was no alternative to dealing with digitalization. Some interview partners expressed the feeling of having been “thrown in the deep end, “which corresponds to the statements from the first interview phase where participants expressed insecurities and resentment toward digitalization. Educators described a decrease in their overall skepticism toward digital formats. Nevertheless, they remained critical and described an increased awareness of what could be reasonably implemented digitally.

*P_03_19: “So I came there (to the QC-session about digitization) feeling a bit unprepared […] because in my personal everyday-life in general practice I really have almost nothing to do with digitization.”*

*P_03_20: “Well, I've always felt a certain 'contra' against digitization because I always think – perhaps unjustifiably – that you could feel too comfortable in this digital world and no longer perceive what is actually really important. But I got used to it […] and then was pushed along by the obligation of having to do it at all, and I'm grateful for that, it was good for me.”*

### Selective coding

**Figure 1:**
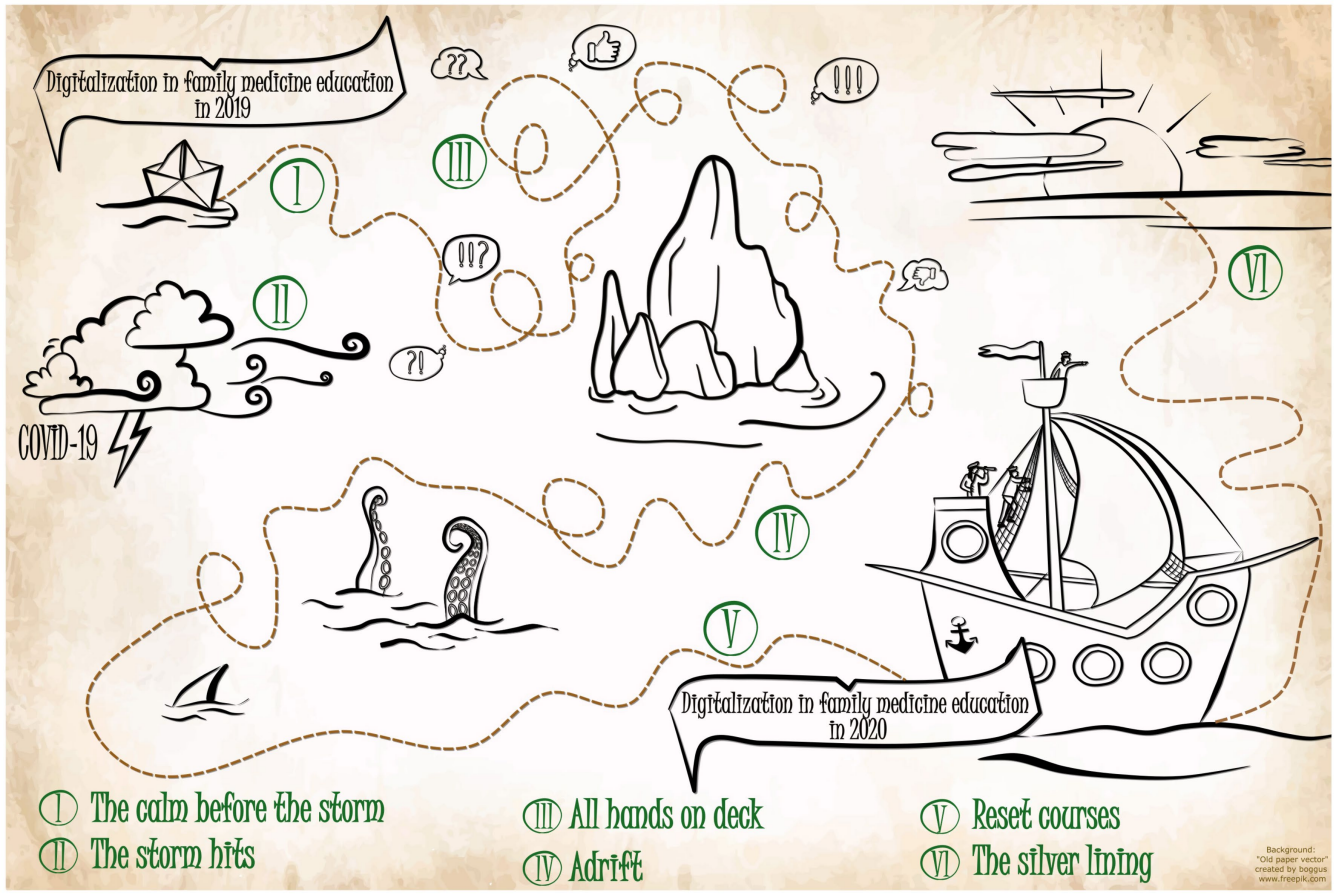
Selective code model “Hold the course(s)”.

The three phenomena described in the axial coding are encompassed by the selective code and visualized in the following model (see Figure 1). It describes the process of transition toward online instruction during the COVID-19 pandemic at the Institute of General Medicine and Interprofessional Health Care at the University of Tübingen from the perspective of the participants of a multiperspective QC on teaching family medicine. It includes six chronological phases, each of which required specific adaptations from individual stakeholders, the organization, and the interactions between them.

#### Stage 1: The calm before the storm

The stakeholders involved in teaching family medicine – students, educators in community-based family medicine practices, lecturers, faculty and staff at the Institute of General Medicine and Interprofessional Health Care – had different attitudes and skill sets concerning digitalization and teaching digital formats. This difference in outlook between students and lecturers was already apparent in the 2019 interviews.

*P_03_19: "I was just impressed by how much input came from the students regarding all these web seminars and formats which I find very exciting but am not familiar with myself."*

*P_01_19: “[…] I found it also became clear that the students are significantly further along in the topic of digitization than the teaching physicians.”*

In 2019, there were only rudimentary approaches to digitizing teaching. Earlier that year, a course was plotted that was meant to ease the idea of digital transformation in family medicine teaching for cautious or inexperienced stakeholders. The goal was to have everybody on board and progress at a velocity that was suitable for stakeholders not yet ready for the digital transformation of teaching.

*P_12_19: "It [digitization] is also something that is still very much in its infancy, at universities in general, and probably also overall.*

*P_04_19: “And what has stuck is that digitization has arrived in very different ways for everyone. […] What I found fascinating was that for some it doesn't play any role at all.”*

Medical educators saw little potential for digital formats in some family medicine courses.

*P_03_19: "Some of the [family medicine courses] have very little to do with digitization. For example, in the physical examination course, the topic of digitization simply doesn't play any role at all.”*

Within the group of teaching physicians, skills and attitudes regarding the digitalization of teaching and digital teaching methods, as well as digital skills in clinical practice, varied:

*P_01_19: "What was interesting, for example, was that the teaching doctors had very different experiences with the topic of digitization in GP practice. For example, […] a practice that is totally paper-based, where really only the billing is done digitally and […] a super modern practice […] "*

Before the first semester under pandemic conditions, only a small-scale and cautious approach was conceivable for the interview partners, and aspects of a broader implementation were not actively considered.

*P_08_19: “I think the topic [digitization] is still relatively far away from actual implementation, though, which makes it difficult to assess now. Just the fact that it is being talked about and seriously considered is a good result.”*

*P_11_20: "Yes, well, I don't think people given it [digitization] much thought before.”*

#### Stage 2: The storm hits

Teaching modes during the summer semester of 2020 were largely dictated by the infection control measures of the COVID-19 pandemic. The pandemic and the related restrictions were a storm that hit stakeholders in the Institute and in teaching practices, like many others around the world, unprepared. The course planned in 2019 had to be abandoned.

*P_02_20: “This phase of uncertainty was then replaced by a phase of action, […] where it was somehow clear that we now had to make the courses digital. […] It was a phase where we […] were under time pressure because digital courses had to be available.”*

The family medicine clinical elective was canceled as there appeared to be no viable adequate digital replacement.

*P_01_20: “[…] We also said that there is just a line […] at the clinical elective which we cannot carry out digitally and we are not allowed to carry it out in-seat, so then we have to drop it.”*

GP teachers had to prioritize their clinical work and fight the pandemic instead of focusing on teaching. Medical students were needed to help in hospitals:

*P_09_20: “Well, Corona meant that teaching came up far short. […] The focus was only on helping out in the hospital and supporting the teams. […] There wasn't so much capacity to turn to things like teaching us students theoretical contents. There were also increased cancellations of seminars [as part of a structured clinical rotation], because they were not digitized immediately.”*

The unpredictability of the pandemic situation and the resulting uncertainty of courses put a strain on students:

*P_11_20: “I found it a pity that as a student you had the feeling that you are a bit on your own or that you have to be somehow open to swift changes and be present all the time because you don't know when it will continue.”*

Furthermore, differences in technical equipment and learning environments made social inequities more visible.

*T_07_20: "And with the students, but also with the educators, this social inequality is also reflected in their housing. If I have small children and I only have a two-room apartment […] or whether I have a four-room apartment or a house where a babysitter can possibly be booked […], that's a very, very huge difference."*

The disparity in terms of technical equipment was even more stark within the group of students, and in some cases surfaced along with significant emotional distress.

*P_07_20: “And those students who did not already have good technical equipment had a big problem. We also received feedback from students, some of whom were quite desperate because they couldn't dial in because they didn't have a stable Internet connection.”*

#### Stage 3: All hands on deck

Due to the pandemic, new concepts had to be developed and implemented with great effort and within a very short time to continue teaching at all.

*P_10_20: "You had to be very flexible, very spontaneous. It was incredibly exhausting to also cover the needs properly. In terms of time and of course in terms of content. I perceived teaching overall to be exhausting and challenging."*

These makeshift solutions then had to be developed further under significant time pressures. This led to uncoordinated, rapid changes in teaching formats, methods, and concepts on previously unknown paths and with sometimes excessive demands on educators. All efforts were made to prevent the teaching and learning ship from sinking, to return to the nautical picture. It was thrown off course.

*P_01_20: "In both cases, I think this semester really required the greatest efforts that have ever been made for teaching by really everyone involved, […] you had to make both the content and the conversion from analogue, or in-seat teaching, to digital in a very short time."*

The course corrections caused stakeholders to find themselves in uncomfortable, previously unknown waters. In the course of the summer semester of 2020, those involved in teaching had to adapt quickly to this new teaching environment. Individual learning processes took place. These included technical skills, such as operating video conferencing software, but also didactic skills, such as moderation of online seminars. Although there were formal training sessions, e.g., on how to operate software, most skills were learned directly within the teaching process in an experiential or self-taught way. In the interviews before the transition, some educators expressed concern that they would not be able to keep up with new digital formats. Due to the transition, educators described a loss of their instructional communication competencies, especially these that characterized and defined them in their educator role.

*P_01_20: What is really completely lost, however, is everything that characterizes me to some extent as a lecturer, that you sometimes make a joke or that you sometimes clown around or something like that, so you can transmit humor quite badly via this medium, unfortunately.*

The realization that there was no viable alternative quickly reduced the initial skepticism toward digital formats.

*P_03_20: "Yes, and with online instruction, I also got to know and appreciate the advantages of it and that was an important thing for me because on my own I wouldn't have dealt with it, I just wouldn't have felt like it."*

#### Stage 4: Adrift

As the first waves of the pandemic receded in July of 2020, these initial learning processes and events were followed by frustration about the compromise or makeshift solutions: while they had fulfilled their initial purpose, in retrospect they turned out to be unsatisfactory as time went by.

*P_08_20: "I am basically still positive, but I also still see many, many aspects from another side, from a rather sobering side."*

For example, the use of digital teaching methods is particularly limited in practical, “hands-on” course contents.

*P_07_20: "We have done these […] complementary care methods completely online but in the long run it is not possible to convey everything that way and maintain the same quality. That's just the way it is. For a short time, there was hope that it might be possible but that has not been fulfilled.*

The clinical elective in the community-based practices were described as offering unique experiences that could not be substituted digitally:

*P_01_20: “We did it in the sense that we offered a digital substitute but nobody can tell me that you can digitally replace the workplace-based experience in the family medicine clinical elective.”*

The rapidity with which the transition took place highlighted the requirements and limitations as well as points of conflict and possibilities to cooperate in digitalization, none of which were mentioned in the first interviews in 2019.

*P_08_20: "The problem is […] the short-term nature […] that put quite a strain on the summer semester […] apt to make you perceive a basically good thing as disadvantageous and difficult, in that you simply have to revise a lot of things in a very short time and perhaps don't find the best solution and there are many uncertainties."*

The QC in family medicine met in July of 2020 and provided an opportunity for an exchange of experiences and evaluation of formats.

*P_01_20: “I think the QC was really useful this semester, especially for this debriefing, in which we collected all perspectives on how this semester was experienced. […] We didn't have a meeting during the Corona period, but the way of thinking, the experience from the previous QCs, has of course influenced me very strongly.”*

Participants shared their frustration about how online instruction limited their repertoire in instructional communication competencies, such as humor.

*P_08_20: "I always like to say that when we talk about it [the online instruction in the first semester under contact restrictions] or things like that: I'm someone who also works with humor and examples and so on, and you can forget that in an online context, it doesn't work.”*

#### Stage 5: Reset course

After the waves had calmed, some educators questioned teaching concepts and contents, including the extent to which digitalization could meaningfully take place in family medicine teaching. At this point, everyone involved refocused on the plotted course and again set sail toward the general direction outlined before the pandemic. Thanks to individual and collective experiences, previously unknown hurdles could now be navigated. Thus, concepts and contents were already evaluated and adapted during the semester.

*P_04_20: "Now we've just done it and it actually worked but now we're learning, […] what we can do better and we don't discuss it for five years beforehand, […] but we do it now and then see what we can do better".*

According to the interview partners, digitalization had changed university teaching and would continue to do so, bringing with it new kinds of challenges.

*P_03_20: "[…] You post a question in the chat room and then it takes a while until someone answers and then I have a single answer from someone and I still don't know how it is with the rest of the group. […] That's a big problem […] that you can't depict in any way. It has something to do with the group experience and also with the possibilities of facial expressions."*

#### Stage 6: The silver lining

The interview partners also saw digital formats as important tools for specific, targeted use in family medicine teaching, complementary to in-seat teaching.

*P_10_20: “You simply have to distinguish between courses that require presence, where you also have to give the student the opportunity to practice and to ask questions directly while practicing. And if you want to impart knowledge, which works very well via theoretical paths and webinars, […] You should weigh the options and split it up if necessary. That's my experience now from the summer semester."*

The experiences during the exclusively digital semester shaped participants’ views on quality management and the evaluation of digital methods. The goal of training family physicians well can continue to be pursued, enriched by experiences that would never have been made without the pandemic situation.

*P_02_20: "In this respect, I believe that by doing everything digitally, it became clear what cannot be done with online instruction. […]. But overall, I think positively and yes, with a few new questions, like ‘How do I ensure quality now?’.“*

## Discussion

By conducting interviews with different stakeholders on teaching and learning in university-based and community-based settings, a model of the digital transformation process of family medicine teaching during the pandemic at the University of Tübingen was developed. The participants of the QC in family medicine teaching found the restrictions imposed by the COVID-19 situation to shape the process of digital transformation of teaching. The pandemic permanently changed both university and community-based family medicine teaching. It also challenged individual stakeholders and their communication, both in class (student-instructor), on the institutional level (instructor-instructor, instructor-course management), and between sectors (university and community-based). The experience-based model allows an analysis of the digital transformation process in family medicine teaching caused by a strong external stimulus. The six stages allow for the following structured comparison of requirements, needs and effects in a reflection of existing literature. Lessons learned are highlighted in Boxes 1–6 after each stage.

### Stage 1: The calm before the storm

Before the pandemic, the participants described the digital teaching methods in family medicine as only available in rudimentary approaches, which corresponded to the general situation throughout Germany (6). GP teachers at the university hospital are primarily physicians who also instruct medical students. They have little or no formal training in medical education since medical didactics training is not mandatory in Germany. The results of this study revealed, in line with previous research, that stakeholders had different levels of prior knowledge and experience with online instruction as well as different attitudes toward it (7, 8).

The educators’ instructional communication competency was mainly derived from personal experiences of in-seat training. They had little or no concept on how to expand to online instruction. Just because digital formats have been growing in popularity for a few years (4, 5) did not mean that all stakeholders shared this interest or were ready to come on board. Surveying the status quo is therefore essential for determining the starting point for further development (45). The cautious, small-step approaches that had been envisioned did not involve community-based teaching. There was neither a focus or common goal for the digitization of teaching nor a clear concept of how to get everyone on board according to their capabilities. These might have helped maintain a more determined course during the following stage.

BOX 1 **Lessons learned in Stage 1 “The calm before the storm”**

- Reflecting on the status quo is essential in order to identify aspects in need of improvement.
- Different stakeholders have different attitudes, experiences, and instructional communication competencies that must be considered.
- Different teaching settings and the unique prerequisites of each setting should be considered.
- The incorporation of individual experiences from in-seat teaching to online instruction needs guidance.

### Stage 2: The storm hits

In Tübingen, the start of the pandemic and the restrictions imposed on faculty and GP practices showed how vulnerable community-based teaching in GP practices was (29): Contact restrictions during the summer semester of 2020 led to a shutdown of the majority of bedside and workplace-based learning opportunities out of concern for patients’ and students’ health, exacerbated by GP teachers’ clinical engagement in the pandemic. Since a digital simulation could not be developed in such a short amount of time without prior planning, in Tübingen the course was substituted with a clinical case report write-up, which all stakeholders found inferior to workplace-based teaching in GP practices. The case write-up did not provide any opportunity for communication exchange between the stakeholders. The preference of bed-side teaching has been described by both educators and medical students, mainly due to personal and emotional engagement and direct feedback (9, 14, 48, 54).

The sudden introduction of digital-only teaching as a reaction to contact restrictions affected stakeholders differently: *Medical students*, while least challenged by new digital tools and most positive toward the methods (14), were affected by the sudden shift toward digital-only teaching and the uncertainty related to their lectures and courses. As other research has shown, participants of our study identified aggravated social disparities for students (37–39) and increased pressures (35, 36).

In contrast to medical students, *medical educators* had a steeper learning curve in terms of digital skills (9). GP teachers especially stated they would not have taken this step without proper cause. GP teachers had to leave their familiar roles and settings and develop new skills to perform confidently in this unfamiliar virtual terrain. Lacking ideas or skills to transfer their educational competencies to virtual classrooms, the learning curve was steepest for them. Bereft of alternatives, they either had to hold fast to the railing or drop out of teaching altogether, which regrettably, some did (55). The metaphor of educators holding on to the railing is significant: even though there was no clear concept of the transformation process to online instruction at the time, these community-based GP teachers were willing to continue working together with medical educators at the institute in the hope that a solution would be found. Trust in the leadership of the teaching organization was a key element.

BOX 2 **Lessons learned in Stage 2 “The storm hits”**

- A strong stimulus can provide tailwinds and direction for the digitalization process of teaching but may lead to reactive measures instead of proactive planning.
- The stimulus affected stakeholders differently but generally diverged their attention from teaching and learning toward other, more immediate goals.
- Hands-on teaching, especially in community-based settings with a loose association with the university, is a vulnerable setting at such times.
- Clear leadership and an associative bond to the teaching organization are protective factors in such a stage.

### Stage 3: All hands on deck

Stakeholders’ reactions toward the ensuing digitalization process of family medicine teaching ranged from anxiety to curiosity and confidence, from initial rejection to gratefulness for the opportunity. Online instruction tools had been available before and during the pandemic. However, very few such instruments were routinely used in medical teaching in Germany. Interview participants stated that, initially, known in-seat formats were simply replaced with digital formats – under the motto ‘same, but digital’. This simple 1:1 conversion from in-seat to digital ensured that teaching did not have to stop altogether. Case reports from other universities confirm this (41, 56). However, too little attention was given to the fact that educational and communicative strategies needed to be adjusted to the digital setting.

Most available research also points out the Herculean task of digitizing available courses (9, 19, 40, 57). Participants reported that the shift to online teaching also comprised changes in their instructor role: In asynchronous formats, new functions such as content creators and curators arose. Instructors shifted to instant messaging communication with students.

In synchronous video formats (such as videoconferences), the shift also challenged their role as instructors and their communication with student groups. In medical workplace-based teaching especially, the value of the teaching physician as a role model has been demonstrated. If a teacher is not able to be eminent and elicit responses in his or her students, learning is not optimal (9). GP teachers’ frustration of not being able to use humor in their digital interactions illustrates the importance of interpersonal components in instructional communication even in medical education.

Stakeholders acknowledged efforts by the university to provide technical and methodical support for online instruction. By having some technical stressors of online teaching alleviated by moderator training, technical instruction, and help in organizing video calls, educators could again focus on their competencies and eminence as physicians and role models.

However, community-based educators expressed regret about insufficient collaboration on didactic concepts and the application of online instruction methods (41). Opportunities for networking (e.g., with other faculty members or educators) both within the Institute and nationally were found lacking, which mirrors existing research (21). The example of Homburg’s approach to digitalization of decentral teaching formats might have helped faculty in Tübingen and led to a much better experience for medical students than writing a case report. In retrospect, a lack of low-threshold, easily accessible cross-regional exchange on digital solutions during the pandemic has become painfully obvious. This exchange of ideas could have facilitated the creation of a network on instructional communication competence for medical educators, which points to a need to address further on a national level in the post-COVID-19 world (13, 41, 58, 59). In our nautical model, there would be not one but a plethora of tiny ships bobbing and floating in treacherous waters, with too little communication between vessels.

BOX 3 **Lessons learned in Stage 3 “All hands on deck”**

- Being forced to try out new practices can reduce inhibitions and prejudices against online instruction.
- Learning by doing works for digitalization, if there is trust in the organization and support available.
- Peer-teaching is a useful and low-threshold option.
- Communication is key, not only within the classroom but also between educators (within faculty), between sectors (university-community) and between faculties – but underdeveloped in Germany.

### Stage 4: Adrift

Medical students in the interviews and other research described the lack of hands-on teaching as the greatest downside of the digital shift (13, 23, 34), highlighting the need for critical evaluation of newly digitized courses (14, 29). From the initial, more reaction-driven stages, participants voiced increasing insecurity and frustration during the third and fourth stages of the transformation, which is consistent with several case reports (2, 21, 40, 60). Many stakeholders’ assumptions about the limitations of online instruction from the first interview phase were confirmed (5, 14, 15, 18, 29). However, the negotiations initiated in these stages also led to a differentiation of ideas of what online instruction could and could not achieve (41). Important aspects of instructional communication like humor or emotional involvement (17) were described as insufficiently addressed.

According to the interview participants, the ensuing frustration was natural and necessary to reassess the current position of online instruction after the first semester. These reflections came naturally due to the significant changes and new experiences and should take place explicitly when implementing courses to align reality with stakeholder perceptions.

When workplace-based learning became feasible again later in the pandemic, experiences made with decentral teaching formats generated new perspectives for such scenarios. For example, synchronous digital seminars enabled course managers to continue to connect learners from various distant learning sites to each other (48) – one learning effect being that decentral teaching could be supplemented but not substituted digitally. Further evaluation of these tools for medical education could contribute to the routine implementation of digital communication channels, enabling remote learning and professional activities across regions in community-based teaching (11).

The key to collecting this information and providing a marketplace for constructive communication and exchange of ideas was, in the case of our institution, a quality circle in community-based teaching in which stakeholders and interview participants participated (46). It allowed integration of perspectives by educators and students and an evaluation of the situation.

BOX 4 **Lessons learned in Stage 4 “Adrift”**

- Experiences made with digital tools change attitudes and behaviors and allow a reassessment of the change processes.
- A substitution of medical teaching for digital formats is not feasible, especially when it comes to bedside teaching in community-based settings.
- Stakeholder frustration with digital tools is an important indicator of what works and what does not – it should be discussed explicitly and with an open mindset.
- Teaching formats should be reflected upon promptly and frequently.
- Community-based teaching can be supplemented by digital means, especially by using digital networking tools to connect community-based teaching sites.
- A multiperspective QC on teaching can provide a forum for such an exchange and for individual efforts to be made visible.

### Stage 5: Reset course

Personal negotiations, the exchange of experiences, and assessments about the digitalization of family medicine teaching in the quality circle led to a consensus about how to continue as an organization. Individual efforts had been made already, but this deliberate discussion, with integration and negotiation of stakeholders’ ideas and experiences in the development of a new course, was important for the subsequent semesters. While this process of realigning the course of the ship was experienced as burdensome, it ultimately led to a reduction in individual skepticism and to an adaptation of teaching to the specific community-based teaching environment. A central exchange of ideas on instructional communication competencies enriched the quality circle participants and facilitated the implementation of communication strategies on the community level.

Educators exhibited similar attitudes as those described by Dorfsman (25) with respect to the different teaching formats. For example, GP teachers craved a return to hands-on bedside teaching in family medicine practices. At the same time, they were pleased with the possibilities that blended learning offered for the physical examination course and teaching across regional distances in a community-based setting. Overall, concerns about trying out the new technical possibilities decreased in all interviewed stakeholders, which mirrors available research (10–12).

The Institute for General Medicine and Interprofessional Health Care was tasked with providing recommendations and best practice examples for digitalizing medical content. They also worked on plotting a new course for subsequent semesters of online instruction, consistently asking the stakeholders about their experiences and incorporating their expectations into new course plans. This “bottom-up” process seems a promising approach in the management of community-based teaching and provided a platform for an exchange about instructional communication (50).

BOX 5 **Lessons learned in Stage 5 “Reset course”**

- The general direction of change processes should build upon concrete stakeholder experiences and should be negotiated proactively.
- Concrete goals should be formulated, consistently expanded, and reflected upon.
- A suitable framework for reflection should be used (e.g., a QC on teaching).

### Stage 6: The silver lining

Participants made it clear that the goal of family medicine teaching remains the same: To train competent physicians to serve the needs of their communities. Unanimously, participants consented that digitalization of family medicine teaching must promote this goal. The wealth of experience gained through the transition has sharpened the focus on digitization. It occurred first in individual stakeholders affected by a strong external stimulus. By trial and error and by sharing insights on methods that could meaningfully contribute to family medicine teaching, the organization as a whole learned. After this process, the participants had a clear vision of digitization in family medicine and regarded it as a meaningful component for the future of community-based teaching. Being forced to leave shallow, well-trodden waters and adapt to a new setting, they also gained new individual competencies in didactics and communication.

Worldwide, COVID-19 had a cataclysmic effect on medical teaching (1–3, 58). Our model, based on different stakeholders’ experiences, can be abstracted and applied to major external influences on teaching in the future. In general, experiences of digital transformation shape attitudes and skills, and vice versa. If a significant need arises without alternatives, even the most cautious in-seat education enthusiast can and will “walk the plank” toward digitalization and benefit from the experience. Students are happy to follow along and get in the boat but emphasize the importance of maintaining a personal touch in their studies. This is particularly critical as it can be assumed to be conducive to learning and a shared goal between educators and students (17).

On the institutional level, important cornerstones have been laid. According to neoinstitutionalist doctrine, large institutions, such as universities, base their actions on legitimacy vis-à-vis their environment and its norms and expectations (61). The shift to digital-only teaching can be seen as a major external factor that could trigger a profound change process (62). At least for university hospital teaching, a number of stakeholders have improved their teaching competencies and developed a more differentiated view of online instruction and its implementation in the medical curriculum (18–22, 41). A more systematic implementation of online instruction in this setting can be expected in the future. For community-based teaching in GP practices, the potential of online instruction has not been fully realized and should remain a focus of future efforts in curricular management and medical education research. At the very least, GP teachers have become more conscious of the fact that they are not only physicians but also educators, with the latter role requiring both a certain skill set and a different mindset. While this may be known on some level, it is too seldom made explicit. For the community-based teaching setting especially, an integrated perspective as provided by the QC in Tübingen seems practical and helpful, both in institutional QM efforts and medical education research.

Another aspect worth considering in the future is the impact of the COVID-19 pandemic on social inequities. This was mentioned by interview partners in our study and has also gained attention among researchers (37–40, 63). This underlines again the need for considering social aspects in transforming medical education – with and without digitalization. A multi-layered, multi-perspective approach as provided by the QC could facilitate an awareness and consideration of social inequities in teaching (64).

BOX 6 **Lessons learned in Stage 6 “The silver lining”**

- The goal of family medicine teaching has remained the same, but the method by which this can be achieved has been adapted.
- In how far digitalization can help achieve this goal has been adapted.
- Individual experiences have contributed to organizational learning processes. Those must now be used to plan ahead and work toward the didactic goal, mindful of the strengths and limitations of digitalization.

## Strengths and limitations

Our study design enables a comprehensive view of different stakeholder viewpoints on the transformation process from in-person to online instruction. In the literature, description of the transition is mainly limited to retrospective analyses and observations (2, 19, 24, 48). The two phases of interviews before and after the transition created a unique data set that depicts the initial situation unbiased by the change process. No data on the transformation process itself was collected in the first interviews. This was only considered retrospectively. This study fills a gap with its before-and-after comparison and the consideration of an integrated view of the stakeholders. To capture a more comprehensive view of online family medical teaching and instructional communication, a follow-up study that adds the after-pandemic perspective to the results described here is needed.

Since the GT method allows for a high degree of flexibility (65), statements that did not play a role in the first interview phase but were mentioned in the second interview phase could be included in the analysis. To generate a dense model of the transformation process, the usual steps, such as analytical induction to test and modify a preliminary hypothesis, were performed consistently on the entire data material (66). Following Glaser (67), the constant comparison was performed for quality assurance and new codings were again compared with already analyzed material to continuously involve all the material in the research process and to be sure that the derived model represented the whole data (68).

Following Malterud’s method to evaluate the sample size of qualitative research, the sample size in this study is small (69). The challenges in recruiting participants during the pandemic were softened by the commitment and existing communication channels of the quality circle for family medicine teaching (46). Nevertheless, conducting interviews during the pandemic was subject to the unpredictability of the pandemic and therefore constantly changing regulations and a generally tense situation. GP teachers especially were challenged by having to maintain patient care in their GP practices. The fact that they responded to our interview invitation shows how motivated the GP teacher participants were. Regarding the experiences of different stakeholders with the transformation processes in family medicine teaching in Tübingen, the analysis yielded a theoretical saturation. Therefore, data collection was terminated after the second interview phase (70). The results are valid for the local transformation process but, despite the local setting, the model shows promising consistency with national and international results. A derivation of general principles for digital transformation is therefore to be seen as limited under the aspect of the local context.

To further increase credibility, especially considering the involvement of the author team in the QC, the research process was continuously documented (68) and presented to uninvolved GP teachers and other researchers in a so-called peer debriefing or member check (68, 71) at conferences (52) and in qualitative methodological workshops, which allowed for some external validation of the results.

## Conclusion

Based on the results and supported by the literature, future digital or mixed-digital projects should be easier and faster to implement. In the current context, the results of this study and the literature suggest retaining in-seat formats, especially to maintain quality in practical, hands-on courses. Thus, in-seat teaching is not replaced but merely complemented by online instruction to offer students the most versatile learning experience possible and support their professional development. Quality management should involve all those involved in teaching at their respective levels of knowledge.

As the likelihood of further extreme pandemics has increased in recent decades (72), we should strive to learn from our experiences of the COVID-19 pandemic. The presented key messages can help navigate upcoming challenges in medical education.

1. Reflecting on the status quo is fundamental in order to identify aspects that need improvement.
2. Despite the same stimulus, the conversion process had different effects on different groups of stakeholders.
3. A strong stimulus to try things out can reduce inhibitions and thus make some individual experiences and learning processes in education possible in the first place. In this context, peer teaching is a useful and low-threshold option.
4. In order not to drift aimlessly, concrete goals should be formulated and consistently expanded upon with reflections. This process should take place in a suitable framework (e.g., QC).
5. The quality of new teaching formats should be reflected upon and adjusted as promptly as possible considering instructional communication.
6. The goal of family medicine teaching has remained the same. The means to achieving it have been expanded on an instructor level by adapting instructional communication competencies and by the new method of online instruction.

There may be formats or methods available that we hesitate to use for teaching. It may behoove all those involved in education to develop the courage and initiative to jump ship on occasion and release new concepts or formats, even when they aren’t fully developed. Instead of waiting for the next external force, like pandemic restrictions, to push us along into treacherous waters, let us plot and chart our own course of travel!

## Author’s note

We use the term “transformation” in this paper to refer to the transition toward using digital media that had been in planning and whose implementation was sped up significantly by external forces, namely the COVID-19 pandemic and related restrictions.

## Data availability statement

The pseudonymized data supporting the conclusions of this article will be made available by the authors, without undue reservation.

## Ethics statement

The studies involving human participants were reviewed and approved by Ethics review board of University of Tübingen (021/2019BO2). The patients/participants provided their written informed consent to participate in this study.

## Author contributions

M-TS: study idea, data collection, qualitative analysis, manuscript draft, and prepared Figure 1. HF: review, editing manuscript, and English proofreading. SJ: project funding, review, and editing manuscript. RK: study idea, qualitative analysis, prepared Figure 1, review, and editing manuscript. All authors contributed to the article and approved the submitted version.

## Conflict of interest

The authors declare that the research was conducted in the absence of any commercial or financial relationships that could be construed as a potential conflict of interest.

## Publisher’s note

All claims expressed in this article are solely those of the authors and do not necessarily represent those of their affiliated organizations, or those of the publisher, the editors and the reviewers. Any product that may be evaluated in this article, or claim that may be made by its manufacturer, is not guaranteed or endorsed by the publisher.
