# Supplementary material for: ‘Hold the course(s)!’ – a qualitative interview study of the impact of pandemic-triggered contact restrictions on online instruction in community-based family medicine teaching
Source: Front Med (Lausanne). 2023 Aug 2;10:1231383. doi: 10.3389/fmed.2023.1231383 (PMC10433760; doi:10.3389/fmed.2023.1231383)
Supplement: Supplementary file 1 [file Data_Sheet_1.docx]

Supplement 1: Outline for the 1^st^ Interview

**Outline for individual interviews for the study "Contributions of an interprofessional quality circle for improving the quality of family medical teaching in Germany".**

Version: Dec 18.2018

| **I. Introduction** |
| --- |
| Introduction of the doctoral student, explanation of the purpose of the study, reference to voluntary nature, recording and data protection and anonymized publication of the data in professional journals. |
| Explanation of the interview procedure |
| **II. Introductory Questions / Warm-Up** |
| What is your experience with the topic of quality assurance in family medical teaching? |
| In the last meeting of the quality circle you dealt with the topic of digitization. How was the topic dealt with in the quality circle? |
| **III. Main Questions** |
| How are you involved in the quality circle? |
| How do the participants work together? |
| How would you describe the process of a Quality Circle meeting? |
| What happens between the sessions? |
| What contributes to a successful session? |
| How would you describe the outcome of the work completed in the last session? |
| What has changed about the Quality Circle since you first participated? |
| How would you describe the effect of the Quality Circle on family medical teaching? |
| **IV. Final Question / Wrap-Up** |
| What else would you like to tell me in this context? |

Thank you for participating!

Supplement 2: Outline for the 2^nd^ Interview

**New outline for individual interviews for the study "Contributions of an interprofessional quality circle for improving the quality of family medical teaching in Germany"**

Version: 24.04.2020

| **I. Introduction** |
| --- |
| Introduction of the doctoral student, explanation of the purpose of the study, reference to voluntary nature, recording and data protection and anonymized publication of the data in professional journals. |
| Explanation of the interview procedure |
| **II. Main Questions** |
| How did you experience teaching in the past summer semester? |
| Last year, you addressed the topic of digitalization in a meeting of the Quality Circle. What do you think about this topic now? |
| How would you describe your attitude towards the digitalization of teaching? |
| Has anything changed in your attitude towards digital teaching in the last year? If yes, what? |
| Have you acquired any skills or knowledge in digital teaching in the last year?  If yes,  Which ones?  How did you acquire these skills or knowledge? |
| How would you describe the effect of the Quality Circle on family medical teaching over the past year? |
| **IV. Final Question / Wrap-Up** |
| What else would you like to tell me in this context? |

Thank you for participating!
